# The Missouri Medical and Surgical Journal

**Published:** 1845-06

**Authors:** 


*The Missouri Medical and Surgical Journal.* May, 1845. Vol. I; No. 1. (In exchange.)

This is the first number of a new journal, monthly, of 24 pages, recently started at St. Louis, and is the second of the kind published in that city. It is not however to be supposed, from this circumstance, that the first has been so successful as to induce others to commence a new one for the profits. The first, the St. Louis Medical and Surgical Journal, was issued under the auspices of those connected with the medical department of the St. Louis University, and this has been commenced in the interest of that branch of Kemper College. Whether the spirit of rivalry will be sufficiently active to long support two journals, remains to be seen. The number before us is handsomely executed, and contains a well written article on the Uses of Iodine, by T. Barbour, M. D., besides other original and selected matter.

